# Ancient DNA reveals early use of melons in China’s Song dynasty

**DOI:** 10.1073/pnas.2610284123

**Published:** 2026-09-04

**Authors:** Nathanael Walker-Hale, Yunfei Zheng, Marion Verdenaud, Lara Pereira, Oscar Alejandro Pérez-Escobar, Michaela Preick, Abdelhafid Bendahmane, Michael V. Westbury, Michael Hofreiter, Hanno Schaefer, Xinyi Liu, Guillaume Chomicki, Susanne S. Renner

**Affiliations:** ^a^ Department of Biosciences, Durham University, Durham DH1 3LE, United Kingdom; ^b^Zhejiang Institute of Archaeology and Cultural Heritage, Hangzhou, Zhejiang Province 310023, China; ^c^ Institute of Plant Sciences Paris-Saclay, Université Paris-Saclay, CNRS, National Research Institute for Agriculture, Food and the Environment, Université d’Evry, Gif-sur-Yvette 91190, France; ^d^ Ecology and Evolutionary Biology, School of Biosciences, University of Sheffield, Sheffield S10 2TN, United Kingdom; ^e^ Royal Botanic Gardens, Kew, London TW9 3AE, United Kingdom; ^f^ Faculty of Mathematics and Natural Sciences, Institute for Biochemistry and Biology, University of Potsdam, Potsdam 14476, Germany; ^g^ Section for Molecular Ecology and Evolution, Globe Institute, University of Copenhagen, København K 1350, Denmark; ^h^ Department of Health Technology, Section for Bioinformatics, Technical University of Denmark, Kongens Lyngby DK-2800, Denmark; ^i^ Department Life Science Systems, Technical University of Munich, Freising 85354, Germany; ^j^ Department of Anthropology, Washington University in St. Louis, St. Louis, MO 63130; ^k^ Department of Biology, Washington University in St. Louis, St. Louis, MO 63130

**Keywords:** *Cucumis melo*, ancient DNA, Song dynasty, melon domestication, archaeogenomics

## Abstract

Melon (*Cucumis melo* L.) domestication is thought to have occurred independently once in Northeast Africa and twice in India, but archaeobotanical seed remains point to a possible additional domestication event in China. Because *Cucumis* seeds are difficult to diagnose morphologically, genomic data from archaeological material are needed to evaluate these scenarios and reconstruct ancient melon traits. We sequenced two Song Dynasty (960–1279 CE) melon seeds from Shuomen Gugang (China), recovering 5.5× and 2.1× nuclear genome coverage. Nuclear and chloroplast analyses place both seeds within cultivated *C. melo* from China, within the “*agrestis*” East Asian gene pool. To assess whether these seeds carried traits associated with sweet dessert melons, we examined loci underlying fruit phenotypes. Neither seed carried alleles for orange flesh; one harbored an allele linked to yellow/orange peel, the other possessed alleles associated with green flesh and reduced acidity. Since wild melons are monoecious, the presence of the derived andromonoecy allele in one seed, associated with rounder fruit shape, suggests early selection on fruit morphology. Together, these findings indicate that Song Dynasty melons were likely consumed as fresh or culinary fruits rather than sweet dessert melons. Their flesh coloration resonates with Song-period aesthetic sensibilities, exemplified by jade-green celadon ceramics frequently crafted in melon-shaped forms. By anchoring East Asian archaeobotanical remains within modern melon genomic variation, this study provides a temporal framework for melon cultivation in China and shows how ancient genomics can illuminate past crop use.

Melon (*Cucumis melo* L.) is an important Cucurbitaceae crop, with worldwide cultivation exceeding 22 million tons in 2024 (1). Melon domestication has been extensively studied over the past decade, yielding valuable insights into the roles of selection and introgression (2–4). How the crop spread across Asia and what its early cultivated forms were like, however, remain poorly documented.

Molecular evidence suggests that melon domestication occurred independently once in Northeast Africa and twice in India (2, 3). Archaeobotanical evidence has hinted at an early cultivation, and possibly independent domestication of *Cucumis* in the lower Yangtze region of China, where seed remains span the Neolithic and Bronze Age (5). Domestication is difficult to infer from seed morphology alone in some genera of Cucurbitaceae, notably *Cucumis*, because seed characters overlap across domesticated, feral, and wild forms, and degradation can obscure diagnostic features (4, 6). Molecular data from archaeological material are therefore necessary to anchor these remains within the genomic diversity of the crop.

The inconsistency between genetic and archaeological evidence points to three possible scenarios: a) early *Cucumis* use in East Asia involving a species other than *C. melo*; b) independent domestication of *Cucumis melo* in ancient China, with descendants persisting among modern Chinese varieties; and c) early Chinese melons are cultivated *C. melo* introduced from the Indian centers of domestication. Given the continuous archaeological record in the Lower Yangtze since 7000 BP, a combined archaeo-genomic approach is timely.

Numerous *Cucumis* seed remains were recovered from archaeological sites across China. We extracted DNA from 24 seeds: 20 from the Nanshan and Tingshan sites in Shaoxing, dating from the Spring and Autumn period (770–476 BC), but we could not recover sufficient endogenous DNA. Four were recovered from Shuomen Gugang (henceforth Gugang), an ancient port at Wenzhou (Fig. 1 *A* and *B*). During the Song Dynasty (960–1279 CE), Gugang served as a major harbor connecting maritime trading systems across the western Pacific and Indian Oceans. Excavations in 2024 revealed an archaeobotanical assemblage comprising abundant rice as well as peach, grape, olive, jujube, and *Cucumis* seeds. Tropical and warm-temperate fruits including lychee and kiwifruit were also present, attesting to extensive maritime trade connections. Two *Cucumis* seeds from waterlogged deposits dating to the Southern Song provided sufficient endogenous DNA (Fig. 1*C*); we radiocarbon dated another seed from the same context to 930–800 cal. BP (c. 1020-c. 1150 CE).

**Fig. 1.**
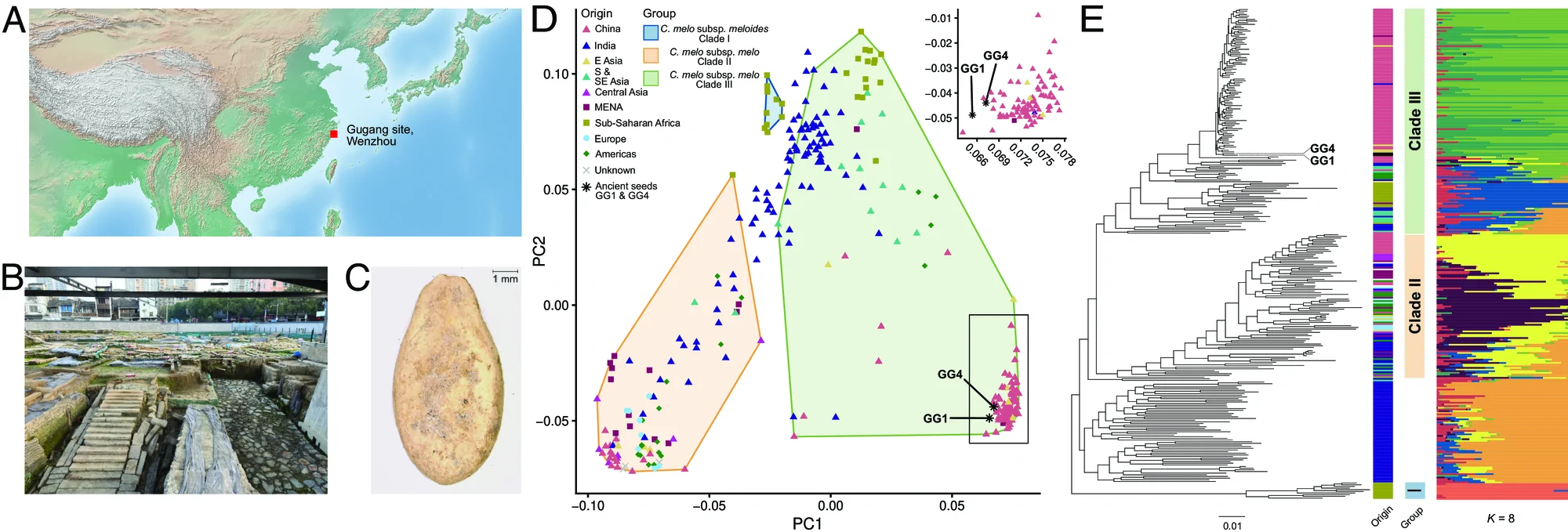
Phylogenomic placement of ancient seeds. (*A*) Location of the Gugang archaeological site in Wenzhou, China. (*B*) The Gugang site. (*C*) Ancient melon seed from Gugang. (*D*) Principal Components Analysis of ancient seed genomes alongside 294 extant melon genotypes, based on inferred genotype likelihoods. (*E*) Maximum likelihood phylogeny of the same genotypes plus 9 outgroups, based on 30,597 fourfold degenerate sites; branch lengths in expected substitutions per site. Tip colors indicate geographic origin and group (*Inset*). Admixture proportions for K = 8 ancestral populations, estimated from genotype likelihoods at positions with no missing data, are aligned to tree tips. For K = 2 to K = 16, see figure S13 in ref. 7.

Here, we generate and analyze ancient DNA from archaeological *Cucumis* seeds from China to i) confirm their taxonomic identity, ii) place them within the genomic diversity of cultivated melon and establish a timestamp for cultivated melon in East Asia and test whether early Chinese material can throw light on melon domestication, and iii) reconstruct the fruit traits and likely uses of ancient Chinese melons.

## Results and Discussion

We performed low-coverage sequencing of 32 libraries prepared from 24 *Cucumis* seeds recovered from three sites in China, alongside extraction and library blanks (average coverage from unique hits 0.00017×, see table S1 in ref. 7). Two seeds from Gugang (GG1 and GG4) yielded sufficient endogenous DNA for deep sequencing. After quality filtering and duplicate removal, we recovered 37 and 15 million unique fragments aligning to the *C. melo* DHL92 reference genome, yielding 5.5× and 2.1× coverage (150× and 73× chloroplast coverage), respectively. Misincorporation patterns confirmed bona fide ancient DNA (see figures S1 and S2 in ref. 7). We combined our ancient data with extant accessions from Zhao et al. (3) to infer pseudohaploid sequences and genotype likelihoods. Principal components analysis and phylogenomic analyses place our ancient seeds closest to cultivated *C. melo* from China in clade III (*agrestis* clade *sensu* 3) (Fig. 1), a result consistent with chloroplast data and robust to missing data (see figures S5–S12 in ref. 7).

Our data confirm that historical East Asian *Cucumis* is indeed *C. melo*, and provides a timestamp for cultivated melon of the East Asian “*agrestis*” lineage in the Lower Yangtze since at least the Song dynasty. Previous research (3) inferred a single domestication event in the *agrestis* clade. As such, our result is consistent with introduction from the broader Asian domestication pool rather than an independent Chinese domestication. However, our data cannot rule out an independent Chinese domestication from a now-extinct or unsampled wild progenitor. No confirmed wild *C. melo* populations are known from China, and further field sampling and genomic data are needed before this hypothesis can be conclusively ruled out.

Beyond the lower Yangtze, morphologically attested *C. melo* has been reported from ancient burials in northwestern China (c. 700 CE) and from tombs of pre-Han (Wuwangdun, 238 BCE) and Western Han royals (Haihunhou, 59 BCE; Mawangdui, c. 168 BCE) in southern China, indicating that cultivated *C. melo* was introduced into China before the Han Dynasty. Given that the earliest records are from southern China contexts rather than from northern sites along the Silk Route, it is plausible that *C. melo* reached southern China before the Han period from South Asia via a network spanning across mountains in southwest China and the Himalayas, historically known as the Tea Horse Route. Subsequently, *C. melo* could have been reintroduced again to China through maritime networks during the Song Dynasty. The relationship between our Song dynasty seeds and Neolithic *Cucumis* (c. 7000–4000 BP) in the lower Yangtze remains unresolved; given the continuity of *Cucumis* in archaeobotanical records spanning the Neolithic and Bronze Age, a local domestication scenario from a gene pool not represented by extant genotypes cannot be ruled out. Further aDNA sampling targeting Neolithic or Bronze Age seed remains would be needed to resolve this question.

**Fig. 2.**
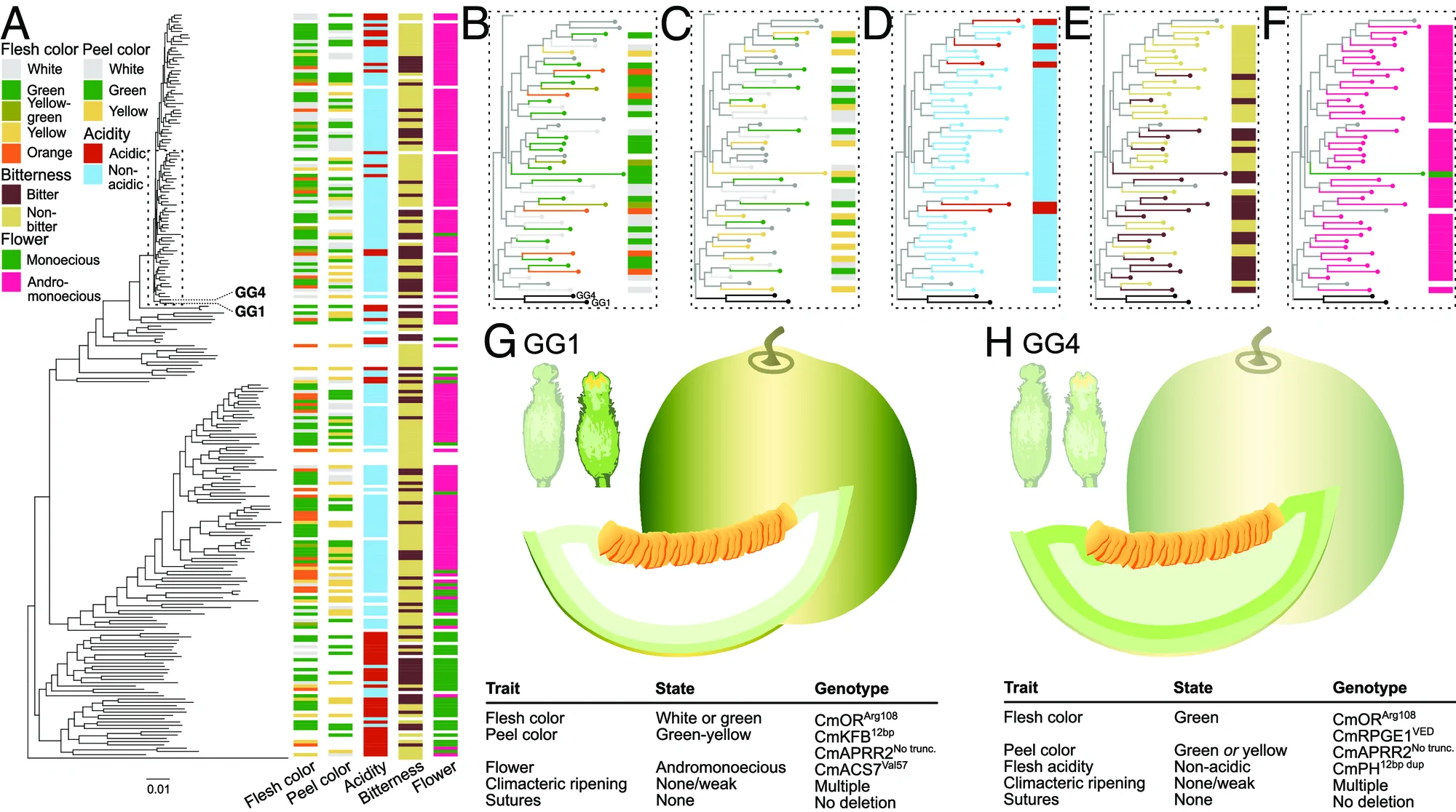
Comparative analysis of ancient fruit phenotypes. (*A*) Distribution of four phenotypes across tips in the inferred tree; samples lacking all trait data were removed. (*B*–*E*) Subtrees surrounding our ancient samples for (*B*) fruit flesh color, (*C*) fruit peel color, (*D*) fruit acidity, (*E*) fruit bitterness, (*F*) flower type. (*G* and *H*) Reconstructed appearance of ancient fruits and flowers based on inferred phenotypes. *Inset* tables below each melon image summarize trait inferences and supporting genotypes; traits not inferred are shown with reduced opacity. Although exact fruit shape and size cannot be inferred, the CmACS7^Val57^ allele is associated with rounder fruits. Not drawn to scale. For read pileups supporting trait inferences, see figure S14 in ref. 7.

To infer potential phenotypes and uses of these ancient melons, we compared alignments at loci governing fruit and flower traits in *C. melo*. *CmOR* is involved in β-carotene biosynthesis; His108 confers orange flesh and is dominant over Arg108 conferring white/green flesh (8). GG1 and GG4 showed evidence of Arg108, and in GG1 the probability of heterozygosity given the coverage is very low (Fig. 2 *G* and *H*). *CmRPGE1* (9) encodes a protein involved in plastid development and chlorophyll maintenance; variation at this locus determines whether nonorange melons develop white flesh or green flesh. GG4 harbored the green flesh allele (Fig. 2*H*), consistent with higher frequencies of green flesh in clade III, but due to low coverage (only two reads) we cannot rule out heterozygosity. We found no evidence of reported truncations in *CmAPRR2*, suggesting dark immature peel (10, Fig. 2 *G* and *H*). GG1 lacked a white-peel-associated deletion in *CmKFB* (11; Fig. 2*G*), suggesting yellow peel. GG1 and GG4 featured genotypes associated with nonclimacteric varieties at four loci involved in climacteric ripening (12), suggesting green-yellow mature peel in GG1 (Fig. 2*G*). The fruits were likely nonsutured (Fig. 2 *G* and *H*, 3). A single read in GG4 supports a 12-bp duplication in *CmPH* controlling fruit acidity (13), suggesting nonacidic flesh, and both samples fall in a clade with a high frequency of nonacidity (Fig. 2*D*). We found no truncations in the bitterness regulator *CmBt*, although bitterness may also be regulated by *CmBt* promoter variation (3). Andromonoecy has been favored during domestication because it is associated with rounder fruits and facilitates breeding (14). GG1 carried the derived Ala57Val allele in *CmACS7*, suggesting it was andromonoecious (Fig. 2*G*, 14). Alignments supporting genotypes are shown in figure S14 of ref. 7. Summaries of allele frequencies in climacteric and nonclimacteric varieties are shown in tables S3–S6 of ref. 7. Several extant melons exhibit the traits inferred from our Song Dynasty seeds: nonorange flesh, often yellow peel, and white to light-green flesh. These are commonly used as fresh, mild fruits, vegetables, or seeds. Analogs include Korean oriental melon (*makuwa*), Japanese pickling melon (*conomon*), South Indian culinary melon (*acidulus*), and Sudanese tibish and seinat (4). This suggest that melons sharing the trait combination observed in our ancient samples (nonorange, white, or potentially green flesh, and green-yellow peel) were likely used in Song Dynasty China as crisp, mildly sweet fruits, fresh or processed, and potentially also as a source of edible seeds, rather than for high sugar content. This interpretation is further supported by cultural considerations: “oriental melon” types with light-green flesh and yellow peel have long held symbolic significance in East Asia, where abundant seeds were associated with fertility and continuity, and green flesh resonated with established aesthetic values. In particular, green celadon ceramics produced in the Lower Yangtze, including Longquan celadon, were frequently fashioned in melon-shaped forms, with glazes echoing the jade-green tones of melon flesh, a typology that became especially prominent during the Song period, and was frequently recovered from Gugang contexts.

## Materials and Methods

Full methods, including sampling, sequencing, read processing, error assessment, and genotype inference following (15), are provided in *SI Appendix*.

## Supplementary Material

Appendix 01 (PDF)

## Data Availability

DNA sequence data have been deposited in GenBank PRJNA1422990 (16). Supplementary figures are available on Figshare (7).
